# A Novel Carbon-Assisted Chemical Vapor Deposition Growth of Large-Area Uniform Monolayer MoS_2_ and WS_2_

**DOI:** 10.3390/nano11092423

**Published:** 2021-09-17

**Authors:** Jeonghwan Bae, Youngdong Yoo

**Affiliations:** 1 Department of Energy Systems Research, Ajou University, Suwon 16499, Korea; qowjdghks123@naver.com; 2 Department of Chemistry, Ajou University, Suwon 16499, Korea

**Keywords:** carbon-assisted CVD, growth mechanism, monolayer, MoS_2_, WS_2_

## Abstract

Monolayer MoS_2_ can be used for various applications such as flexible optoelectronics and electronics due to its exceptional optical and electronic properties. For these applications, large-area synthesis of high-quality monolayer MoS_2_ is highly desirable. However, the conventional chemical vapor deposition (CVD) method using MoO_3_ and S powder has shown limitations in synthesizing high-quality monolayer MoS_2_ over a large area on a substrate. In this study, we present a novel carbon cloth-assisted CVD method for large-area uniform synthesis of high-quality monolayer MoS_2_. While the conventional CVD method produces thick MoS_2_ films in the center of the substrate and forms MoS_2_ monolayers at the edge of the thick MoS_2_ films, our carbon cloth-assisted CVD method uniformly grows high-quality monolayer MoS_2_ in the center of the substrate. The as-synthesized monolayer MoS_2_ was characterized in detail by Raman/photoluminescence spectroscopy, atomic force microscopy, and transmission electron microscopy. We reveal the growth process of monolayer MoS_2_ initiated from MoS_2_ seeds by synthesizing monolayer MoS_2_ with varying reaction times. In addition, we show that the CVD method employing carbon powder also produces uniform monolayer MoS_2_ without forming thick MoS_2_ films in the center of the substrate. This confirms that the large-area growth of monolayer MoS_2_ using the carbon cloth-assisted CVD method is mainly due to reducing properties of the carbon material, rather than the effect of covering the carbon cloth. Furthermore, we demonstrate that our carbon cloth-assisted CVD method is generally applicable to large-area uniform synthesis of other monolayer transition metal dichalcogenides, including monolayer WS_2_.

## 1. Introduction

Two-dimensional (2D) materials have attracted much attention due to their novel physical and chemical properties [1,2,3,4,5]. Graphene, the most studied 2D material, is thin, flexible, remarkably strong, and has exceptionally high electron mobility and thermal conductivity, allowing for a wide range of novel applications [1,2,6]. However, graphene has a zero bandgap, which results in very low on-off ratios in its applications of electronic devices such as transistors [4,5,7]. On the other hand, unlike graphene, transition metal dichalcogenides (TMDCs) have been intensively studied as new 2D layered materials because they have a sizable bandgap and interesting electronic and optical properties [3,4,5,7]. MoS_2_, a family of TMDCs, has been used as a building block for 2D field-effect transistors due to its high carrier mobility and excellent on-off ratios [8,9,10]. In addition, due to its exceptional physicochemical properties, 2D MoS_2_ has been extensively used for novel 2D electronics, flexible optoelectronics, and efficient catalysis [11,12,13,14,15]. When MoS_2_ is thinned down to a monolayer, its electronic structure and physical symmetries are radically altered, resulting in new physical behavior such as indirect to direct bandgap transitions [16,17,18]. In addition, monolayer MoS_2_ exhibits strong light–matter interactions due to its planar exciton confinement effect [16,19,20]. To increase the potential use of monolayer MoS_2_ in various applications, it is highly desirable to develop methods for preparing monolayer MoS_2_ [8,10,11,15,21,22]. The most well-known mechanical exfoliation method is suitable for producing high-quality single crystalline MoS_2_ flakes, but it cannot control the number of layers of the flakes and is unscalable for mass production [23,24,25,26]. In contrast, the chemical vapor deposition (CVD) method can control the number of MoS_2_ layers and enables wafer-scale synthesis [27,28,29]. However, the conventional CVD method using MoO_3_ and S powder has a problem in that thick MoS_2_ films are formed in the center of the substrate and only MoS_2_ monolayers are generated at the edge of the thick MoS_2_ films [30,31,32,33,34].

This problem is related to the growth mechanism of MoS_2_ in the conventional CVD method. The growth of MoS_2_ is mainly achieved by the reaction of S with suboxide MoO_3-*x*_ species produced from MoO_3_ powder [35,36,37]. MoO_3-*x*_ is highly volatile and improves the reaction kinetics for the formation of monolayer MoS_2_ [35,37]. Monolayer MoS_2_ can be effectively formed when the degree of MoO_3-*x*_ formation is sufficiently high, whereas thick MoS_2_ films are generated when the degree of MoO_3-*x*_ formation is low. Therefore, keeping the degree of MoO_3-*x*_ formation high in the reaction process is a key condition for large-area uniform growth of high-quality monolayer MoS_2_ without forming thick MoS_2_ films. To achieve the large-area growth of monolayer MoS_2_, various methods have been reported, including confined-space CVD, reverse-flow chemical vapor epitaxy, inorganic vapor CVD, and metal organic CVD, etc. [38,39,40,41,42,43,44].

In this study, we report a novel carbon cloth-assisted CVD method that uniformly produces high-quality monolayer MoS_2_ over a large area on a substrate without forming thick MoS_2_ films. As-synthesized monolayer MoS_2_ was characterized in detail by Raman/photoluminescence (PL) spectroscopy, atomic force microscopy (AFM), and transmission electron microscopy (TEM). We reveal the detailed growth process of monolayer MoS_2_ initiated from MoS_2_ seeds by conducting a series of experiments with varying reaction times. In addition, we show that the CVD method employing carbon powder instead of carbon cloth also enables large-area growth of monolayer MoS_2_, confirming the large-area growth of monolayer MoS_2_ by the carbon cloth-assisted CVD method is mainly due to reducing properties of the carbon material, rather than the effect of covering the carbon cloth. Furthermore, we confirm that the carbon cloth-assisted CVD method can be used for the synthesis of other monolayer TMDCs such as monolayer WS_2_.

## 2. Materials and Methods

### 2.1. Conventional CVD Method for MoS_2_ and WS_2_ Synthesis

**Figure 1:**
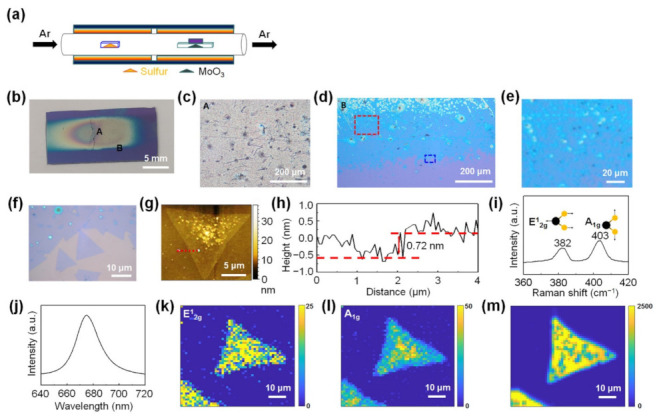
Conventional chemical vapor deposition (CVD) growth of MoS_2_. (**a**) Schematic illustration of the experimental setup for the conventional CVD growth of MoS_2_. (**b**) Optical image of the MoS_2_ synthesized on an SiO_2_/Si substrate using the conventional CVD method. (**c**) Magnified optical image of the region A in (**b**). (**d**) Magnified optical image of the region B in (**b**). (**e**) Magnified optical image of the dotted red rectangle in (**d**). (**f**) Magnified image of the dotted blue rectangle in (**d**). (**g**) Atomic force microscopy (AFM) height image of monolayer MoS_2_ synthesized using the conventional CVD method. (**h**) Height line profiles along the dotted red line in (**g**). (**i**) Raman and (**j**) photoluminescence (PL) spectra of monolayer MoS_2_. (**k**,**l**) Raman maps of the E_2g_ mode and A_1g_ mode of MoS_2_, respectively. (**m**) PL map of monolayer MoS_2_.

MoS_2_ was synthesized by a CVD method using a two-zone horizontal hot-wall tube furnace equipped with a mass flow controller and a vacuum pump (Edwards Vacuum, west Sussex, United Kingdom). The synthetic scheme is illustrated in Figure 1a. In a 1-inch diameter quartz tube, S powder (0.1 g, Sigma–Aldrich, St. Louis, MO, USA, 99.999%) in an alumina boat was placed upstream, and MoO_3_ powder (0.03 g, Sigma-Aldrich, 99.5%) in an alumina boat was put downstream. The growth promoter solution that was prepared by supersaturating NaCl in ethanol was dropped on a clean 300-nm SiO_2_/Si substrate and dried. NaCl serves as a promoter for the growth of MoS_2_ [45]. Na+ in NaCl can react with MoO_3-_*_x_* to form eutectic intermediates possessing a low melting point, promoting the growth of monolayer MoS_2_. The 300-nm SiO_2_/Si substrate was placed face down on the alumina boat containing MoO_3_ powder. We used a vacuum pump to lower the pressure of the quartz tube to 5-mTorr or less to remove air in the quartz tube before the reaction. After turning off the vacuum pump, Ar gas (ultra-high purity, 99.999%, Dong-A Gases, Seoul, Korea) flowed at a rate of 100 sccm until reaching atmospheric pressure. After the pressure reached the atmospheric pressure, Ar gas flowed at a rate of 10 sccm. The temperatures of S and MoO_3_ powder were independently controlled in two separate heating zones. The MoO_3_ powder was heated to 740 °C for 15 min at a rate of ≈47.6 °C min^–1^ and maintained at 740 °C for 20 min. The S powder was heated to 210 °C for 19 min at a rate of ≈9.7 °C min^–1^ and maintained at 210 °C for 16 min. After the end of the reactions, the furnace lid was opened to cool the furnace rapidly to room temperature.

For the synthesis of monolayer WS_2_, S powder (0.3 g, Sigma–Aldrich, 99.999%) and WO_3_ powder (0.05 g, Sigma–Aldrich, 99.9%) were used as precursors. The c-cut sapphire substrate was placed face down on an alumina boat containing WO_3_ powder. After the quartz tube was evacuated to 5-mTorr or less, Ar and H_2_ gases flowed at a rate of 140 sccm and 20 sccm, respectively, and the chamber pressure was maintained at ≈1.6 Torr. The WO_3_ powder was heated to 950 °C for 30 min at a rate of ≈30.8 °C/min and kept at 950 °C for 20 min. The S powder was heated to 210 °C for 32 min at a rate of ≈5.8 °C min^–1^ and maintained at 210 °C for 18 min.

### 2.2. Carbon Cloth-Assisted CVD Method for Monolayer MoS_2_ and WS_2_ Synthesis

The synthesis conditions of the carbon cloth-assisted CVD method are the same as those of the conventional CVD method described above, except that carbon cloth is placed on top of MoO_3_ and WO_3_ powder in an alumina boat for the synthesis of monolayer MoS_2_ and WS_2_, respectively.

### 2.3. Carbon Powder-Assisted CVD Method for Monolayer MoS_2_ Synthesis

The synthesis conditions of the carbon powder-assisted CVD method are the same as those of the conventional CVD synthesis method described above, except that activated carbon powder is mixed with MoO_3_ powder. The mixing ratios of MoO_3_ powder to activated carbon powder used in each experiment were 1:1, 1:2, 1:3, 1:4, 1:5, and 1:10, respectively.

### 2.4. Characterization

Raman spectra and maps were obtained using a 532-nm laser with 100 μW focused through a 100× objective at room temperature. Scanning electron microscopy (SEM) images and energy-dispersive X-ray spectroscopy (EDS) data were taken at 5 kV using a JSM-7900F (JEOL) microscope operating from 1 to 15 kV (JEOL Ltd., Tokyo, Japan). AFM measurement was performed in noncontact mode on an Anton–Paar Tosca 400 AFM instrument (Anton Paar, Sumida, Austria). TEM measurements were performed using a JEM-2100F microscope (JEOL Ltd., Tokyo, Japan).

## 3. Results and Discussion

### 3.1. Thick MoS_2_ Films and Monolayer MoS_2_ Synthesized Using the Conventional CVD Method

Figure 1a shows a schematic illustration of the experimental setup for the conventional CVD method in which solid powders (MoO_3_ and S powder) are used as precursors for MoS_2_ synthesis. Figure 1b shows an optical image of MoS_2_ synthesized on a 300-nm SiO_2_/Si substrate using the conventional CVD method. Figure 1c is a magnified optical image of region A in Figure 1b, showing the MoS_2_ grown in the form of a thick film in the center of the substrate. Figure 1d is a magnified optical image of region B in Figure 1b, showing the edge region of the thick MoS_2_ films. Figure 1e,f show magnified optical images of the dotted red rectangle and dotted blue rectangle in Figure 1d, respectively, confirming the partial formation of monolayer MoS_2_ at the edge of the thick MoS_2_ film. Additional data on the optical characterization of the MoS_2_ grown using the conventional CVD method are shown in Supplementary Materials Figure S1. The growth of such thick MoS_2_ films and partial formation of monolayer MoS_2_ have been commonly observed in MoS_2_ growth by the conventional CVD method using MoO_3_ and S powder as precursors [30,31,32,33,34]. An AFM height image of monolayer MoS_2_ shows small particles grown nonuniformly on the surface of monolayer MoS_2_ (Figure 1g). The line profile obtained along the dotted red line in Figure 1g shows that the thickness of the synthesized monolayer MoS_2_ is ~0.72 nm, which is consistent with the reported thickness of monolayer MoS_2_ (Figure 1h) [19,46].

Raman and photoluminescence (PL) analyses were performed on the as-synthesized monolayer MoS_2_. The Raman spectrum of monolayer MoS_2_ shows that the frequency difference between the E^1^_2g_ mode located at 382 cm^−1^ and the A_1g_ mode located at 403 cm^−1^ was approximately 21 cm^−1^ (Figure 1i), which is consistent with that of the reported monolayer MoS_2_ [31]. Due to the direct bandgap of monolayer MoS_2_, the PL spectrum of MoS_2_ shows a strong A exciton peak at 1.84 eV (Figure 1j) [19,47]. Raman and PL maps show that monolayer MoS_2_ exhibited nonuniform Raman and PL peak intensities, confirming that monolayer MoS_2_ had nonuniform optical and electronic properties (Figure 1k–m). Even when H_2_ was used as a carrier gas with Ar for the MoS_2_ synthesis, thick MoS_2_ films were formed in the center of the substrate and some flakes of monolayer MoS_2_ were partially formed at the edge of the thick films (Figure S2).

### 3.2. Monolayer MoS_2_ Synthesized Using Carbon Cloth-Assisted CVD Method

**Figure 2:**
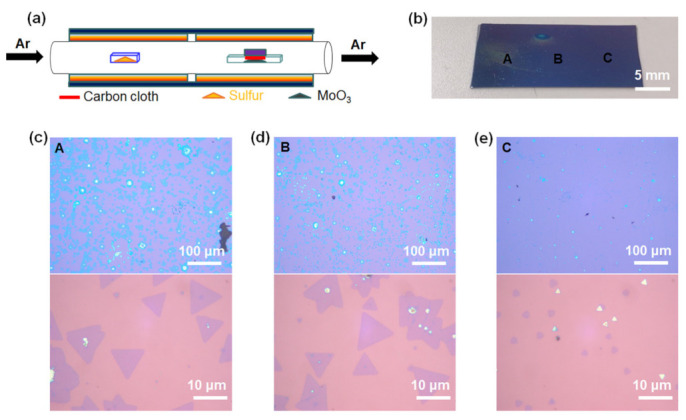
Carbon cloth-assisted CVD growth of monolayer MoS_2_. (**a**) Schematic illustration of the experimental setup for carbon cloth-assisted CVD growth of monolayer MoS_2_. (**b**) Optical image of monolayer MoS_2_ grown on an SiO_2_/Si substrate. Low-magnification and high-magnification optical images of (**c**) region A, (**d**) region B, and (**e**) region C in (**b**).

Figure 2a shows a schematic illustration of the carbon cloth-assisted CVD growth of monolayer MoS_2_. The experimental conditions were the same as those of the conventional CVD synthesis growth, except that the MoO_3_ powder contained in the alumina boat was covered with carbon cloth. Unlike the conventional CVD method, this carbon cloth-assisted CVD method enables the growth of monolayer MoS_2_ without forming thick films in the center of the substrate. Figure 2b is an optical image showing MoS_2_ grown on a 300-nm SiO_2_/Si substrate by the carbon cloth-assisted CVD method, confirming that there were no thick films in the center of the substrate. Figure 2c–e show the low-magnification and high-magnification optical images for regions A, B, and C in Figure 2b, respectively, confirming that monolayer MoS_2_ grew relatively uniformly throughout the substrate without forming thick MoS_2_ films in the center of the substrate. Additional data on the optical characterization of the MoS_2_ grown using the carbon-assisted CVD method are shown in Figures S3 and S4. In addition, we observed that the size of the monolayer MoS_2_ decreased when we moved from region A to region C in Figure 2b. The change in the size of the monolayer MoS_2_ can be explained as follows; on regions A and B located upstream, a sufficient amount of S vapor reacts with MoO_3-*x*_ to form large monolayer MoS_2_, whereas on region C located downstream, the amount of S vapor reaching region C is relatively small, resulting in relatively limited reactions with S vapor and MoO_3-*x*_.

**Figure 3:**
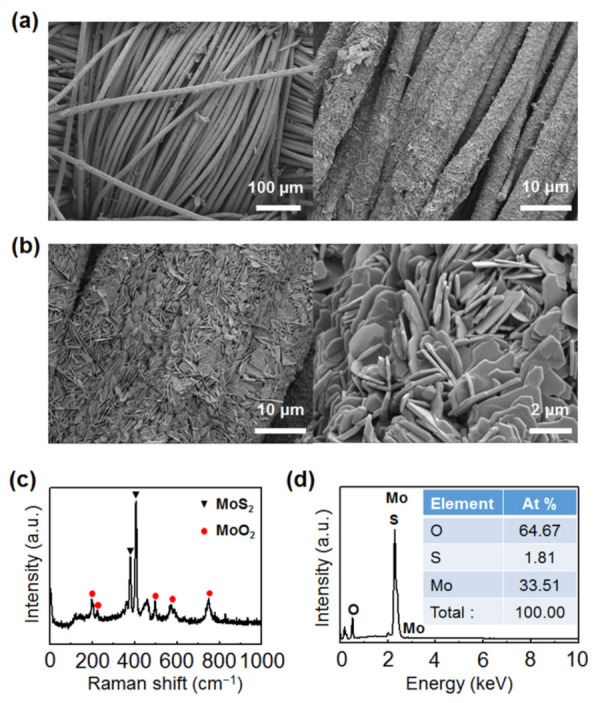
MoO_2_-MoS_2_ nanoplates grown on carbon cloth after carbon cloth-assisted CVD growth. (**a**,**b**) Low-magnification and high-magnification scanning electron microscopy (SEM) images of MoO_2_-MoS_2_ nanoplates grown on carbon cloth after the carbon cloth-assisted CVD growth. (**c**) Raman spectrum of MoO_2_-MoS_2_ nanoplates. (**d**) SEM–energy-dispersive X-ray spectroscopy (SEM–EDS) data of MoO_2_-MoS_2_ nanoplates.

To investigate the mechanisms of the carbon cloth-assisted CVD growth of monolayer MoS_2_, materials formed on carbon cloth during the growth were analyzed. Figure 3a,b show low-magnification and high-magnification SEM images of carbon cloth obtained after the carbon cloth-assisted CVD growth, respectively, confirming that the surface of the carbon cloth was entirely covered with nanoplates with a size of two to three microns. Raman analysis confirms that these nanoplates consisted of MoS_2_ and MoO_2_ (Figure 3c) [48]. In addition, EDS analysis shows that the nanoplates were composed of Mo, S, and O, and the proportion of O was very large compared to the proportion of S (Figure 3d), which confirms that the nanoplates were mostly composed of MoO_2_ and were partially composed of MoS_2_. MoO_2_ is a byproduct that is frequently formed in the conventional CVD growth of MoS_2_ using MoO_3_ and S powder as precursors. MoO_2_ is nonvolatile and has a high melting point, so it remains once it is formed on the substrate. One of the important roles of carbon cloth in carbon cloth-assisted CVD growth is to prevent MoO_2_ from forming on the SiO_2_/Si substrate by allowing MoO_2_ to form on the carbon cloth (Figure S5). Another role of carbon cloth is to improve the reaction kinetics for MoS_2_ growth by facilitating the formation of suboxide MoO_3-*x*_ species formed from MoO_3,_ as carbon acts as a reducing agent.

**Figure 4:**
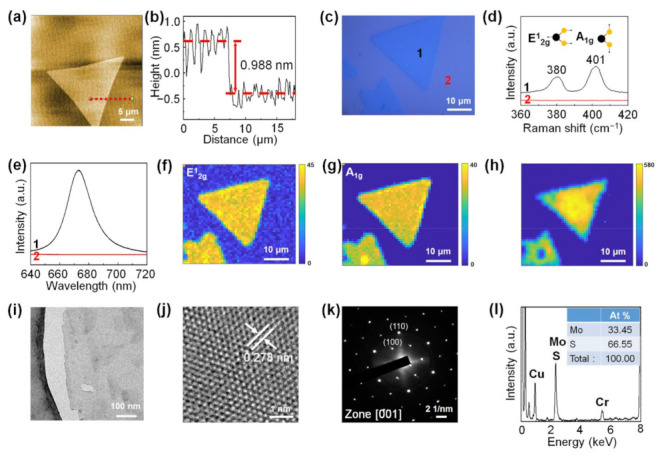
Detailed analysis of monolayer MoS_2_ synthesized by CVD growth using carbon cloth. (**a**) AFM image of monolayer MoS_2_. (**b**) Height line profile along the dotted red line in (**a**). (**c**) Optical image of monolayer MoS_2_. (**d**) Raman spectra taken at points 1 and 2 of (**c**). (**e**) PL spectra taken at points 1 and 2 of (**c**). (**f**,**g**) Raman maps of the E_2g_ mode and A_1g_ mode of MoS_2_, respectively. (**h**) PL map of monolayer MoS_2_. (**i**) Low-magnification transmission electron microscopy (TEM) image of monolayer MoS_2_. (**j**) High-resolution TEM (HRTEM) image of monolayer MoS_2_. (**k**) Selected area electron diffraction (SAED) patterns of monolayer MoS_2_. (**l**) TEM–energy-dispersive X-ray spectroscopy (TEM–EDS) data of monolayer MoS_2_.

Figure 4a is an AFM image of the monolayer MoS_2_ synthesized using the carbon cloth-assisted CVD method, which shows that the surface of monolayer MoS_2_ was clean without any particles, unlike monolayer MoS_2_ synthesized using the conventional CVD method. The line profile obtained along the dotted red line in Figure 4a shows that the thickness of the synthesized monolayer MoS_2_ was ~0.988 nm, which is consistent with the reported thickness of monolayer MoS_2_ (Figure 4b) [19,46]. Additional AFM data of monolayer MoS_2_ are shown in Figure S6. Figure 4c shows an optical image of monolayer MoS_2_ grown on an SiO_2_/Si substrate. The Raman spectrum (Curve 1) of monolayer MoS_2_ taken at point 1 shows the Raman peaks of the E^1^_2g_ mode located at 380 cm^−1^ and the A_1g_ mode located at 401 cm^−1^ (Figure 4d) [31]. Curve 2 in Figure 4d shows the Raman spectrum obtained from the substrate at point 2. The PL spectrum (Curve 1) of monolayer MoS_2_ shows a strong peak at 1.84 eV (Figure 4e), which is consistent with the A exciton peak due to the direct bandgap of monolayer MoS_2_ [19,47]. Curve 2 in Figure 4e is the PL spectrum obtained from the substrate at point 2. Raman and PL maps of MoS_2_ show that monolayer MoS_2_ exhibited uniform Raman and PL peak intensities, confirming that monolayer MoS_2_ had a uniform chemical composition and electronic structure (Figure 4f–h). Figure 4i shows a TEM image of monolayer MoS_2_. The high-resolution TEM (HRTEM) image (Figure 4j) and corresponding selected area electron diffraction (SAED) patterns (Figure 4k) with [001] zone axis confirm the hexagonal lattice structure with the lattice spacing of 0.278 nm assigned to the (100) planes of MoS_2_. In addition, TEM–EDS analysis shows that the monolayer MoS_2_ consisted of Mo and S, and the ratio of Mo to S elements was 1:2 (Figure 4l). The Cu peak originated from the TEM grid, and the Cr peak came from the pole pieces of the TEM.

**Figure 5:**
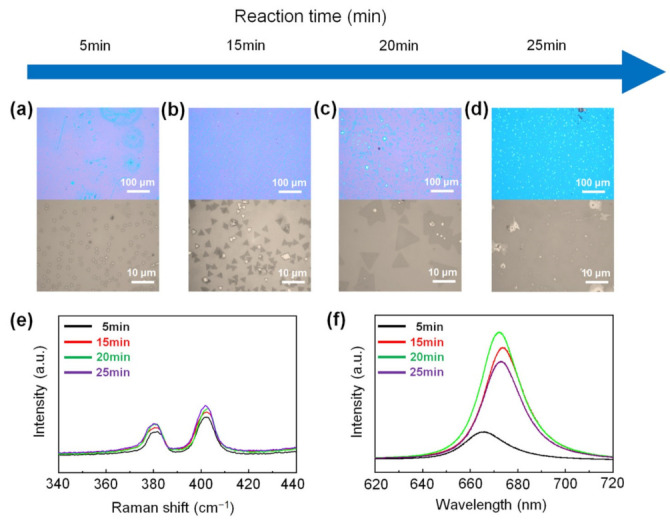
Growth process of monolayer MoS_2_ in the carbon cloth-assisted CVD growth. Low-magnification and high-magnification optical images of monolayer MoS_2_ synthesized at reaction times of (**a**) 5 min, (**b**) 15 min, (**c**) 20 min, and (**d**) 25 min, respectively. (**e**) Raman and (**f**) PL spectra of monolayer MoS_2_ synthesized at reaction times of 5 min, 15 min, 20 min, and 25 min, respectively.

Figure 5 shows the growth process of monolayer MoS_2_ depending on the reaction time in carbon cloth-assisted CVD growth. The reaction time was set to 5, 15, 20, and 25 min, respectively. Figure 5a–d show the low-magnification and high-magnification optical images of MoS_2_ flakes synthesized at each reaction time. At the reaction time of five minutes, small round-shaped MoS_2_ seeds were formed (Figure 5a). At the reaction times of 15 and 20 min, triangular monolayer MoS_2_ was generated, and its size increased with increasing reaction time (Figure 5b,c). Size distribution of monolayer MoS_2_ synthesized at reaction times of 5 min, 15 min, and 20 min is shown in Figure S7. At the reaction time of 25 min, monolayer MoS_2_ films were formed (Figure 5d). The growth of monolayer MoS_2_ depending on the reaction time can be explained as follows. In the initial stage of the reaction (reaction time: five minutes), S vapor and MoO_3-*x*_ vapor are supplied on the substrate to form small MoS_2_ seeds. As the reaction time increases (reaction time: 15 and 20 min), MoS_2_ seeds form on the substrate and grow to form triangular monolayer MoS_2_, and as the reaction time increases, the size of the monolayer MoS_2_ increases. When the reaction time is further increased (reaction time: 25 min), S vapor and MoO_3-*x*_ vapor are continuously supplied to grow triangular monolayer MoS_2_ to form monolayer MoS_2_ films. Figure 5e shows the Raman spectra of MoS_2_ synthesized at each reaction time, confirming that the flakes and films synthesized at all reaction times were composed of MoS_2_. Figure 5f shows the PL spectra of MoS_2_ formed at each reaction time, confirming that all MoS_2,_ except for the MoS_2_ seeds formed at the reaction time of five minutes, exhibited a strong A exciton peak at 1.84 eV, indicating that the as-synthesized MoS_2_ flakes and layers were monolayers. We believe that the variation of the PL peak position originated from the variation of strain or defects of the as-synthesized monolayer MoS_2_ [49].

### 3.3. Monolayer MoS_2_ Synthesized Using the Carbon Powder-Assisted CVD Method

The MoS_2_ synthesis was conducted using the carbon powder-assisted CVD method to determine whether the large-area growth of monolayer MoS_2_ without forming thick MoS_2_ films is because carbon acts as a reducing agent or because carbon cloth physically covers the MoO_3_ precursor. For carbon powder-assisted CVD synthesis, experiments were conducted by mixing MoO_3_ powder and carbon powder in ratios of 1:1, 1:2, 1:3, 1:4, 1:5, and 1:10, respectively.

**Figure 6:**
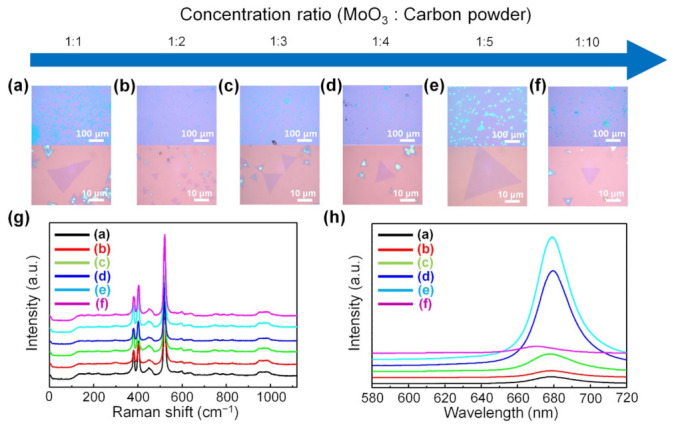
Carbon powder-assisted CVD growth of monolayer MoS_2_. Low-magnification and high-magnification optical images of monolayer MoS_2_ synthesized depending on the mixing ratio of carbon powder to MoO_3_ powder; (**a**) 1:1, (**b**) 1:2, (**c**) 1:3, (**d**) 1:4, (**e**) 1:5, and (**f**) 1:10, respectively. (**g**) Raman and (**h**) PL spectra of monolayer MoS_2_ synthesized using the carbon powder-assisted CVD method.

Figure 6a–f shows low-magnification and high-magnification optical images of monolayer MoS_2_ synthesized with various mixing ratios of carbon powder to MoO_3_ powder, confirming that monolayer MoS_2_ was grown on the substrate over the large area without forming thick films in the center of the substrate. We demonstrated that the carbon material, acting as a reducing agent, plays an important role in the large-area uniform synthesis of monolayer MoS_2_. When the mixing ratio of MoO_3_ powder to carbon powder was 1:1, the MoS_2_ had a nonequilateral triangle shape, which means that MoS_2_ has low crystallinity (Figure 6a). This is because when the ratio of carbon powder is low, the degree of the formation of suboxide MoO_3-*x*_ species formed during the reaction process is low, so the reaction kinetics deteriorate. When the mixing ratio of the MoO_3_ powder to the carbon powder was from 1:2 to 1:10, MoS_2_ with an equilateral triangle shape and high crystallinity was formed. Among them, the largest monolayer MoS_2_ was obtained when the mixing ratio of MoO_3_ powder to carbon powder was 1:5 (Figure 6e). When the mixing ratio of the MoO_3_ powder to the carbon powder was further changed to 1:10, the size of the monolayer MoS_2_ became small (Figure 6f).

The Raman spectra confirm that all synthesized flakes exhibited Raman peaks at the E^1^_2g_ mode and the A_1g_ mode of MoS_2_ (Figure 6g). Figure 6h shows the PL spectra of the MoS_2_ synthesized with various mixing ratios of carbon powder to MoO_3_ powder. As the ratio of carbon powder increased, monolayer MoS_2_ with higher crystallinity was produced, which showed higher PL intensity. The PL spectra of the MoS_2_ show strong A exciton peaks at 1.84 eV when the mixing ratio of MoO_3_ powder to carbon powder was 1:4 and 1:5, confirming that the as-synthesized MoS_2_ flakes were high-quality MoS_2_ monolayers. However, when the ratio of carbon powder to MoO_3_ powder is too high, MoO_3_ is reduced to suboxide MoO_3-*x*_ species and further reduced to form MoO_2_ or Mo, which rather hinders the growth of monolayer MoS_2_. Thus, under this condition, the size of the monolayer MoS_2_ became smaller again and the PL intensity decreased.

We performed the synthesis of monolayer MoS_2_ using graphite powder mixed with MoO_3_ powder (Figure S8). Like the activated carbon powder-assisted CVD method, the graphite powder-assisted CVD method led to the synthesis of monolayer MoS_2_ over a large area on the substrate. These results confirm that the reducing property of carbon is the main factor inducing the large-area growth of monolayer MoS_2_.

### 3.4. Growth Mechanism of Monolayer MoS_2_ in the Carbon-Assisted CVD Growth

During the carbon-assisted CVD growth of monolayer MoS_2_, MoO_3_ is reduced by carbon to form volatile suboxide MoO_3−x_ species, which are further sulfurized to form MoS_2_ on an SiO_2_/Si substrate. The proposed reaction mechanism is as follows [50,51].

2MoO_3_ + *x*C → 2MoO_3−*x*_ + *x*CO_2_ (1)

2MoO_3−*x*_ + (7 − *x*)S → 2MoS_2_ + (3 − *x*)SO_2_ (2)

In this paper, we showed that the reaction kinetics for the growth of monolayer MoS_2_ can be improved by using carbon materials. When no carbon materials were used, thick MoS_2_ films were formed in most areas on the substrate and Figure S1), whereas when carbon materials were used, monolayer MoS_2_ was formed in most areas on the substrate (Figure 2, Figure 6 and Figure S3). Thus, we believe that the carbon materials improve the reaction kinetics for the growth of monolayer MoS_2_ and suppress the formation of thick MoS_2_ films.

The generally accepted mechanism for the growth of monolayer MoS_2_ involves the nucleation of tiny suboxide MoO_3-*x*_ seeds on the substrate surface followed by subsequent sulfurization of these seeds and subsequent growth of monolayer MoS_2_ [50]. Thus, suboxide MoO_3-*x*_ species play a key role in the growth of monolayer MoS_2_. By using carbon cloth or carbon powder, we effectively increased the degree of the formation of suboxide MoO_3-*x*_ species, leading to the growth of monolayer MoS_2_ in most areas on the substrate. On the other hand, the formation of thick MoS_2_ films can be achieved by either the direct nucleation of nonvolatile MoO_3_ or MoO_2_ clusters on the substrate followed by subsequent sulfurization.

### 3.5. Application to Other TMDCs

**Figure 7:**
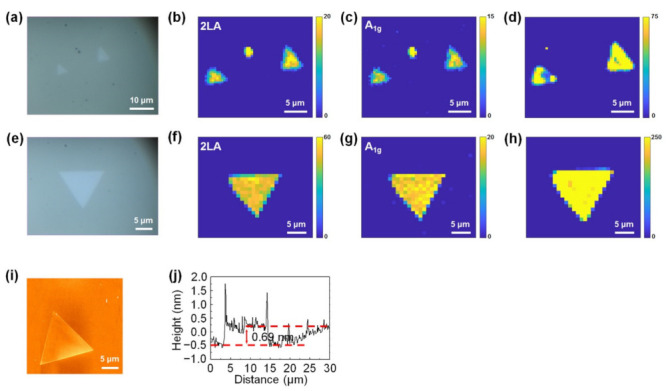
Conventional CVD growth and carbon cloth-assisted CVD growth of monolayer WS_2_. (**a**) Optical image of monolayer WS_2_ synthesized using the conventional CVD method. (**b**,**c**) Raman maps of monolayer WS_2_ synthesized using the conventional CVD method, taken at the 2LA mode and the A_1g_ mode of WS_2_, respectively. (**d**) PL map of monolayer WS_2_ synthesized using the conventional CVD method. (**e**) Optical image of monolayer WS_2_ synthesized using the carbon cloth-assisted CVD method. (**f**,**g**) Raman maps of monolayer WS_2_ synthesized using the carbon cloth-assisted CVD method, taken at the 2LA mode and A_1g_ mode of WS_2_, respectively. (**h**) PL map of monolayer WS_2_ synthesized using the carbon cloth-assisted method. (**i**,**j**) AFM image and height line profiles of monolayer WS_2_ synthesized using carbon cloth-assisted CVD method.

In addition, to confirm that the carbon cloth-assisted CVD method applies to the synthesis of other monolayer TMDCs, we performed the synthesis of monolayer WS_2_ using the conventional CVD method and the carbon cloth-assisted CVD method, respectively. Figure 7a is an optical image of the monolayer WS_2_ synthesized using the conventional CVD method. The size of the monolayer WS_2_ was as small as four microns, and its shape was not an equilateral triangle. Raman and PL mappings at the 2LA mode and A_1g_ mode of WS_2_ show that the monolayer WS_2_ exhibited nonuniform Raman and PL peak intensities, confirming that the monolayer WS_2_ had nonuniform optical and electronic properties (Figure 7b–d). Figure 7e is an optical image of monolayer WS_2_ synthesized using the carbon cloth-assisted CVD method. The size of the monolayer WS_2_ was approximately 13.5 microns, and its shape was an equilateral triangle. Raman and PL mappings at the 2LA mode and A_1g_ mode of WS_2_ show that the monolayer WS_2_ exhibited uniform Raman and PL peak intensities, confirming that monolayer WS_2_ had a uniform chemical composition and electronic structure (Figure 7f–h). Figure 7i shows an AFM image of the monolayer WS_2_ synthesized using the carbon cloth-assisted CVD method, confirming that the surface of the monolayer WS_2_ was clean without any particles. The line profile shows that the thickness of the monolayer WS_2_ was ~0.69 nm, which is consistent with the reported thickness of the monolayer WS_2_ (Figure 7j) [52,53].

The growth of high-quality monolayer WS_2_ by the carbon cloth-assisted CVD method can be explained as follows. For the synthesis of monolayer WS_2_, WO_3_ powder was used as a precursor. The WO_3_ has a significantly high melting point (1473 °C) and its vapor pressure is very low at the reaction temperature (950 °C). Thus, the conventional CVD method produces small monolayer flakes of WS_2_ with very low coverage on the substrate (Figure S9). When carbon cloth is placed on top of WO_3_ powder, carbon acts as a reducing agent and increases the degree of the formation of suboxide WO_3-*x*_ species to improve the reaction kinetics for the formation of monolayer WS_2_. Thus, under this condition, triangular monolayer WS_2_ with increased size forms uniformly on the substrate (Figure S9). Consequently, we confirmed that the carbon cloth-assisted CVD method is generally applicable to the synthesis of high-quality monolayer WS_2_.

## 4. Conclusions

We developed a novel carbon-assisted CVD method for large-area uniform growth of high-quality monolayer MoS_2_. Using the carbon cloth-assisted CVD method, we synthesized high-quality monolayer MoS_2_ uniformly over a large area on the substrate without forming thick MoS_2_ films. Through detailed analyses of the carbon cloth that was used in the reaction and experiments with varying reaction times, we revealed the mechanisms for the large-area growth of high-quality monolayer MoS_2_. In addition, we showed that the carbon powder-assisted CVD method also produces high-quality monolayer MoS_2_ over a large area on the substrate. This confirms that the uniform large-area growth of MoS_2_ using the carbon cloth-assisted CVD method is mainly due to the reducing properties of the carbon material. Furthermore, we demonstrated that the carbon cloth-assisted CVD method can be generally used to synthesize monolayer WS_2_.

## Supplementary Materials

The following are available online at https://www.mdpi.com/article/10.3390/nano11092423/s1, Figure S1: Additional optical characterization of the MoS_2_ grown using the conventional CVD method, Figure S2: MoS_2_ synthesized by the CVD method using H_2_ as a carrier gas with Ar, Figure S3: Additional optical characterization of the MoS_2_ grown using the carbon cloth-assisted CVD method, Figure S4: MoS_2_ flakes grown using the carbon cloth-assisted CVD growth, Figure S5: MoO_2_-MoS_2_ nanoplates grown after the carbon cloth-assisted CVD growth and after the conventional CVD growth, Figure S6: Additional AFM data of monolayer MoS_2_ synthesized using the carbon cloth-assisted CVD method, Figure S7: Size distribution of monolayer MoS_2_ synthesized at reaction times of 5 min, 15 min, and 20 min using the carbon cloth-assisted CVD method, respectively, Figure S8: Graphite powder-assisted CVD growth of monolayer MoS_2_, Figure S9: Monolayer WS_2_ synthesized on a c-cut sapphire substrate using the conventional CVD method and the carbon cloth-assisted CVD method.
