# Supplementary material for: A Novel Carbon-Assisted Chemical Vapor Deposition Growth of Large-Area Uniform Monolayer MoS2 and WS2
Source: Nanomaterials (Basel). 2021 Sep 17;11(9):2423. doi: 10.3390/nano11092423 (PMC8468553; doi:10.3390/nano11092423)
Supplement: Supplementary file 1 [file nanomaterials-11-02423-s001.zip › nanomaterials-1364733-supplementary.pdf]

Supplementary Materials

A Novel Carbon-Assisted Chemical Vapor Deposition Growth of Large-Area Uniform Monolayer MoS_2 and WS_2

Jeonghwan Bae [1] and Youngdong Yoo [2,*]

[1] Department of Energy Systems Research, Ajou University, Suwon 16499, Korea; qowjdghks123@naver.com
[2] Department of Chemistry, Ajou University, Suwon 16499, Korea
* Correspondence: yyoo@ajou.ac.kr

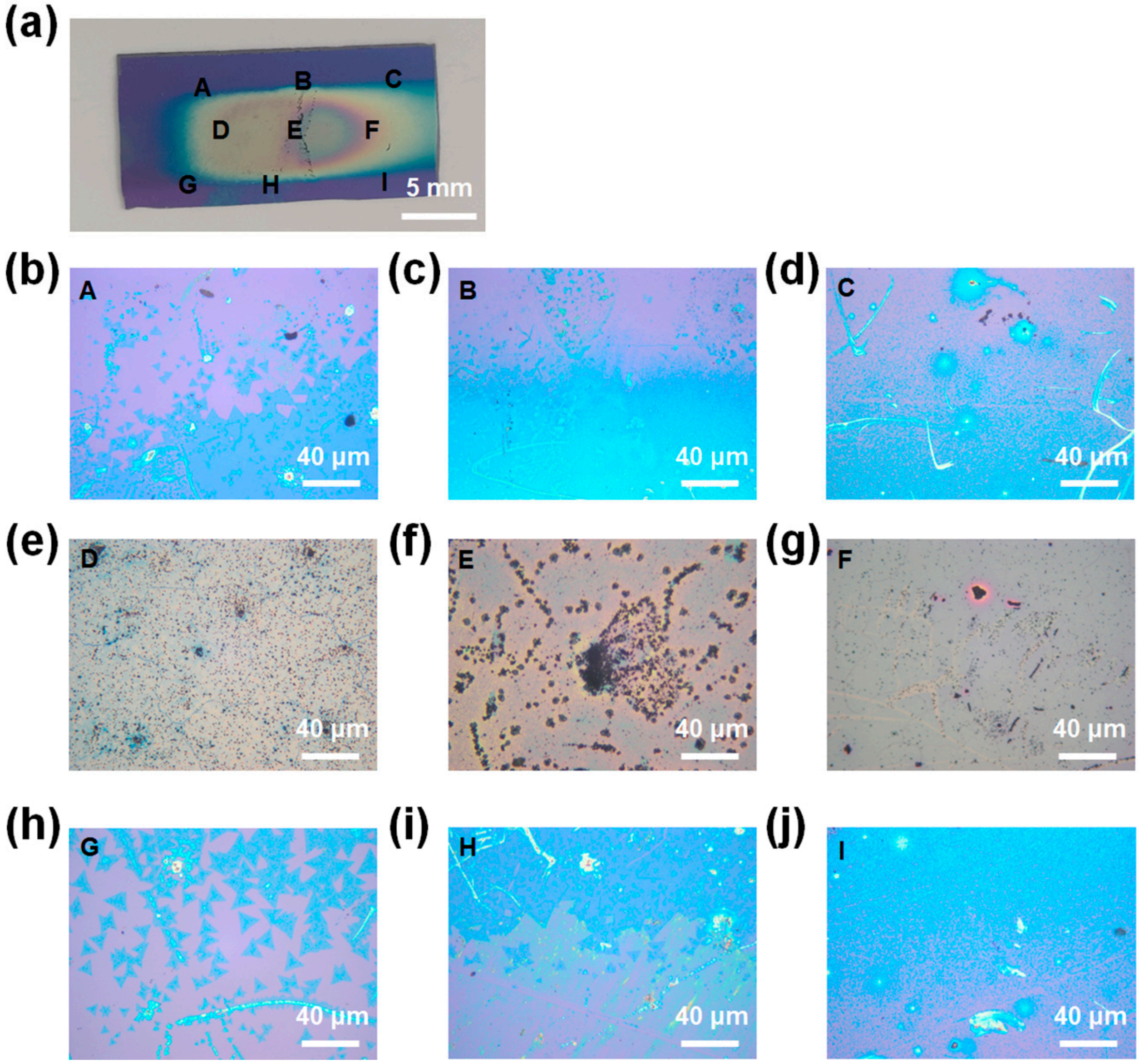

Figure S1. Additional optical characterization of the MoS_2 grown using the conventional CVD method. (a) Optical image of thick MoS_2 films and monolayer MoS_2 synthesized on a SiO_2/Si substrate using the conventional CVD method. (b-j) Magnified optical images of the region A, B, C, D, E, F, G, H, and I in (a), respectively.

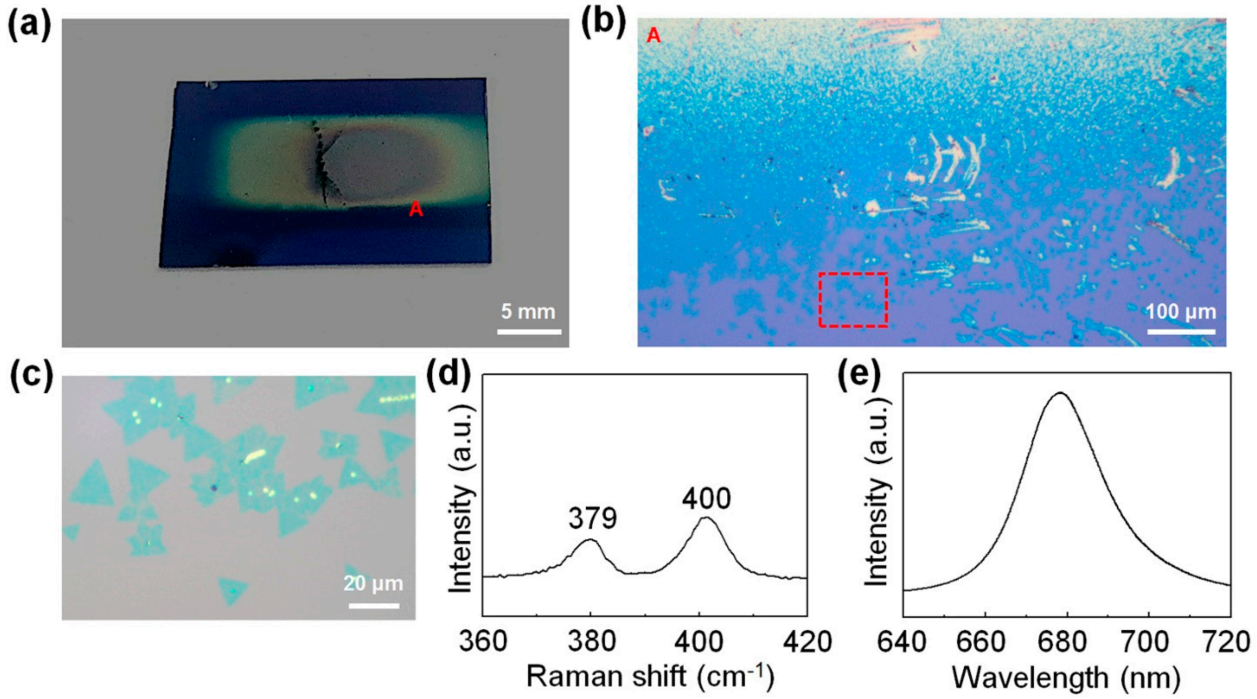

Figure S2. MoS_2 synthesized by the CVD method using H_2 as a carrier gas with Ar. (a) Optical image of the sample. (b) Magnified optical image of the region A of (a). (c) Magnified optical image of the dotted red rectangle in (b). (d) Raman spectrum and (e) PL spectrum of monolayer MoS_2 synthesized partially at the edge of the thick MoS_2 films.

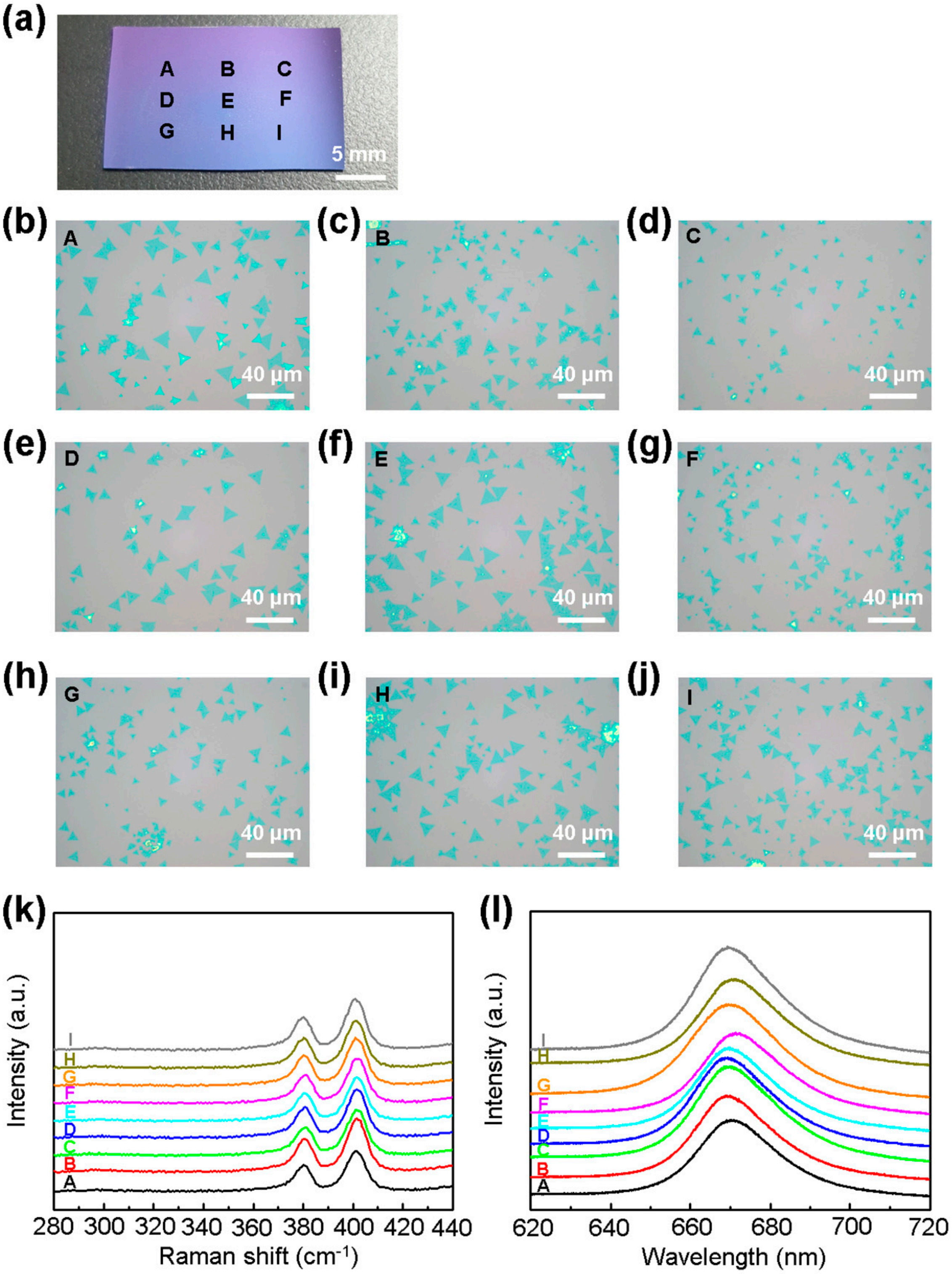

Figure S3. Additional optical characterization of the MoS_2 grown using the carbon cloth-assisted CVD method. (a) Optical image of monolayer MoS_2 synthesized on a SiO_2/Si substrate using the carbon cloth-assisted CVD method. (b-j) Magnified optical images of the region A, B, C, D, E, F, G, H, and I in (a), respectively. (k) Raman and (l) PL spectra of monolayer MoS_2 synthesized in the region A, B, C, D, E, F, G, H, and I in (a).

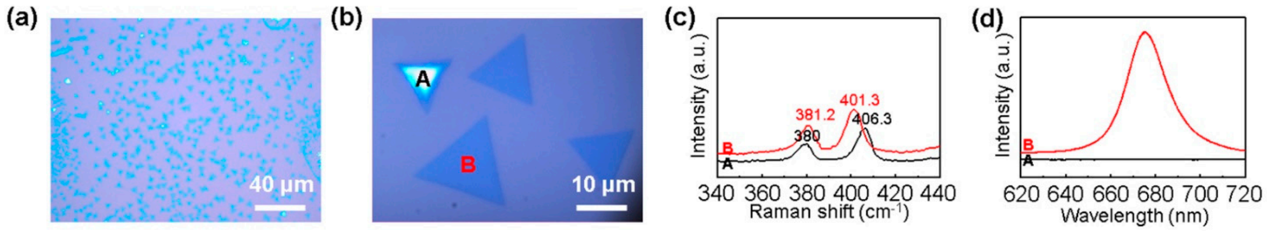

Figure S4. MoS_2 flakes grown using the carbon cloth-assisted CVD growth. (a) Low-magnification optical image of MoS_2 flakes. (b) High-magnification optical image of MoS_2 flakes. (c) Raman spectra taken at the point A and B of (b), respectively. (d) PL spectra taken at the point A and B of (b), respectively. These results show that the carbon cloth-assisted CVD growth produces mainly monolayer MoS_2 over a large area on the substrate and forms partially some MoS_2 multilayer. We believe that the partial formation of some MoS_2 multilayers is due to the local variation of experimental conditions on the substrate.

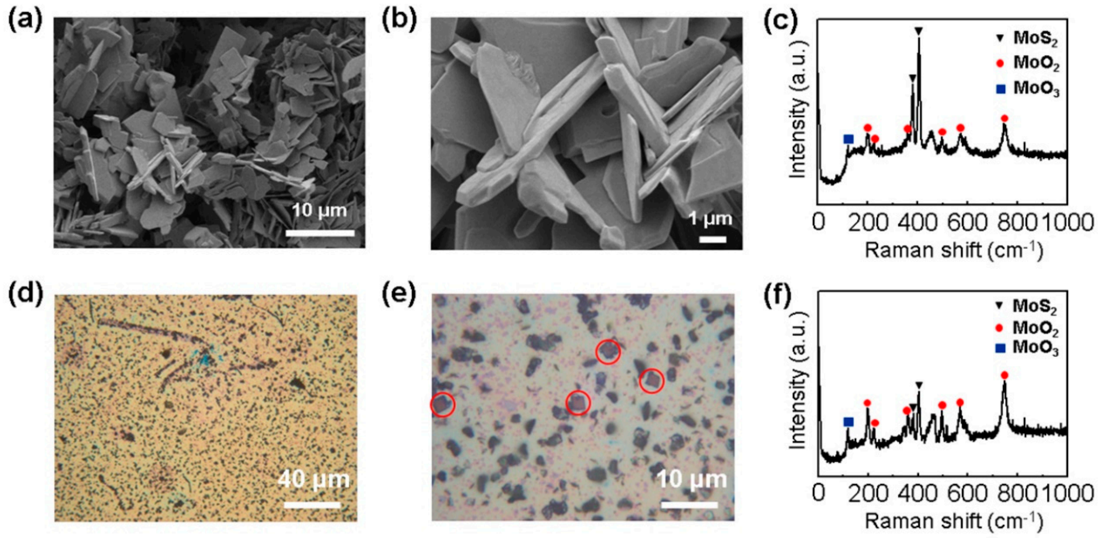

Figure S5. MoO_2-MoS_2 nanoplates grown after the carbon cloth-assisted CVD growth and after the conventional CVD growth. (a,b) Low-magnification and high-magnification SEM images of MoO_2-MoS_2 nanoplates grown on carbon cloth after the carbon cloth-assisted CVD growth. (c) Raman spectrum of MoO_2-MoS_2 nanoplates grown on the carbon cloth. (d,e) Low-magnification and high-magnification optical images of MoO_2-MoS_2 nanoplates (indicated by red circles) grown on a SiO_2/Si substrate after the conventional CVD growth. (f) Raman spectrum of MoO_2-MoS_2 nanoplates grown on the SiO_2/Si substrate. MoO_2-MoS_2 nanoplates were grown on carbon cloth instead of the SiO_2/Si substrate when the carbon cloth is used, whereas MoO_2-MoS_2 nanoplates were grown on the SiO_2/Si substrate when no carbon cloth is used.

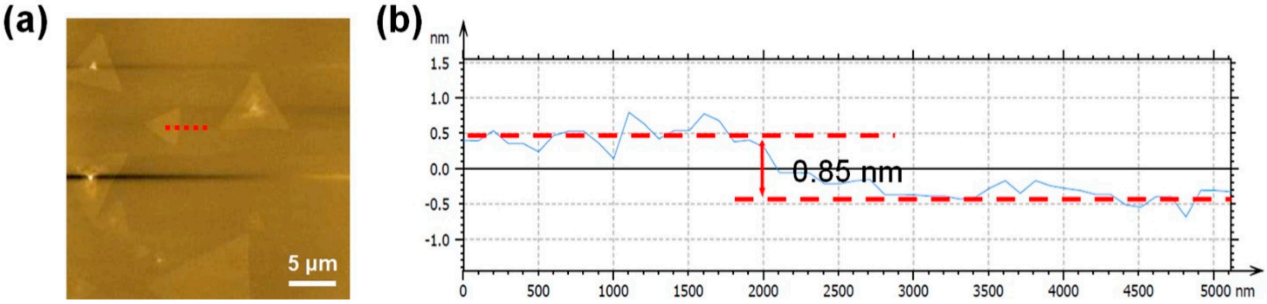

Figure S6. Additional AFM data of monolayer MoS_2 synthesized using the carbon cloth-assisted CVD method. (a) AFM height image of monolayer MoS_2. (b) Height line profiles along the dotted red line in (a).

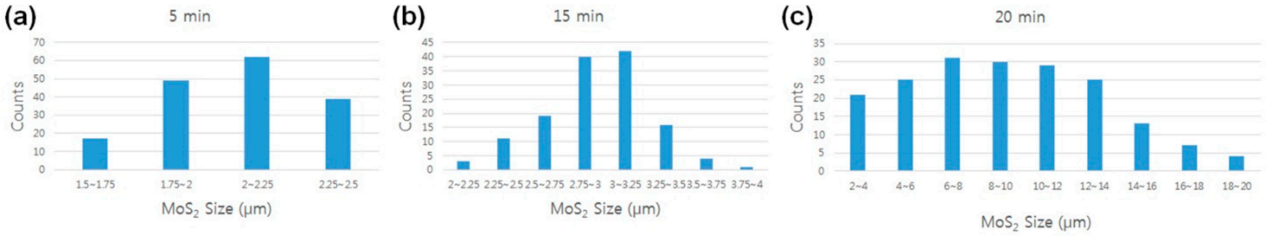

Figure S7. Size distribution of monolayer MoS_2 synthesized at reaction times of 5 min, 15 min, and 20 min using the carbon cloth-assisted CVD method, respectively.

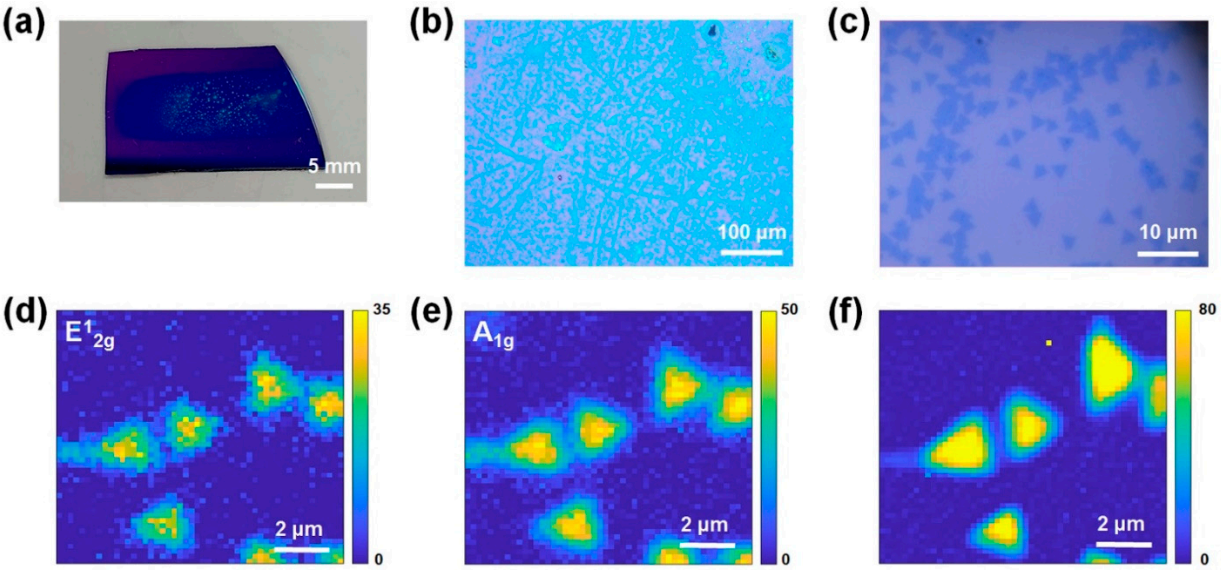

Figure S8. Graphite powder-assisted CVD growth of monolayer MoS_2. (a) Optical image of the sample. (b) Low-magnification and (c) high-magnification optical images of the monolayer MoS_2. (d,e) Raman maps of the E_{2g} mode and A_{1g} mode of MoS_2, respectively. (f) PL map of monolayer MoS_2.

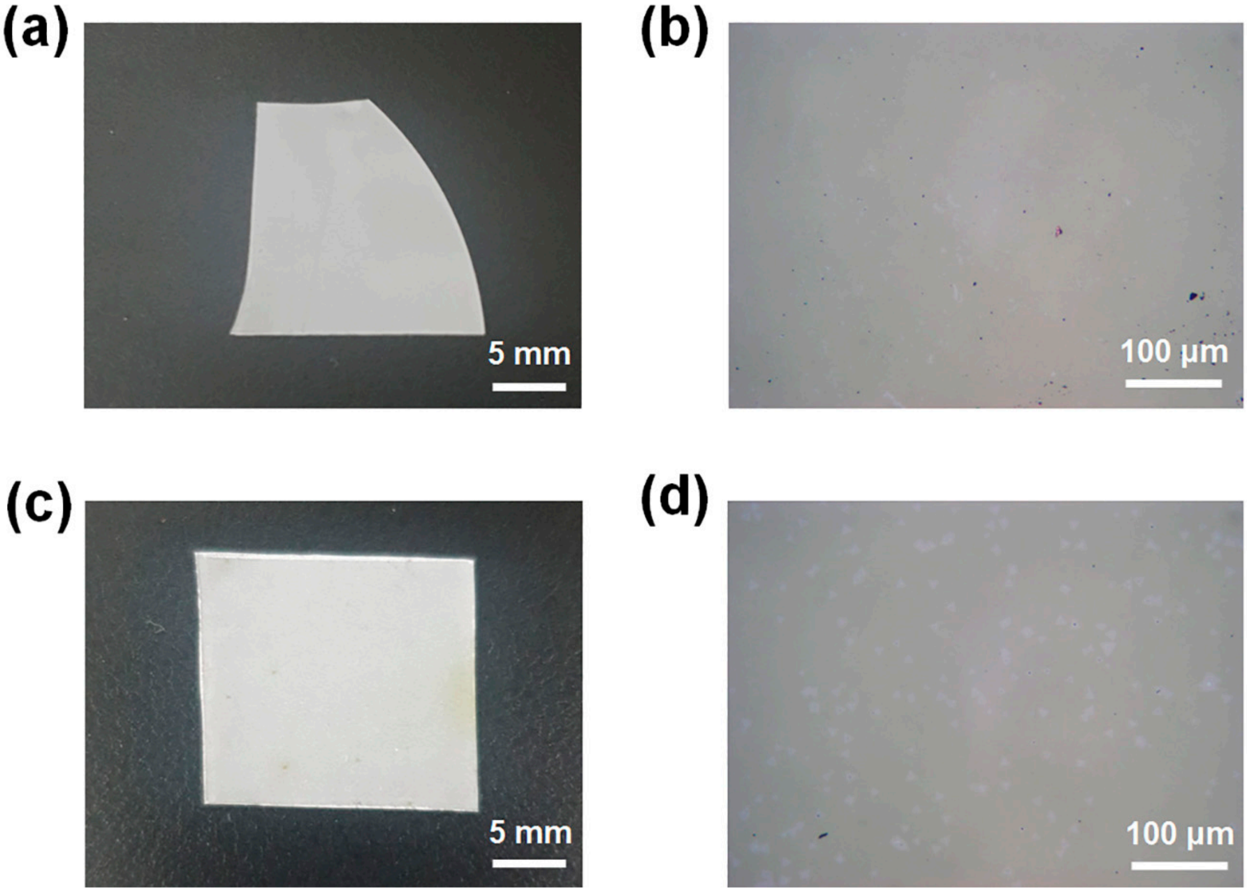

Figure S9. Monolayer WS_2 synthesized on a c-cut sapphire substrate using the conventional CVD method and the carbon cloth-assisted CVD method. (a) Optical image and (b) magnified optical image of the WS_2 synthesized on a c-cut sapphire substrate using the conventional CVD method. (c) Optical image and (d) magnified optical image of the WS_2 synthesized on a c-cut sapphire substrate using the carbon cloth-assisted CVD method.
